# Functional analysis of cytosolic tryparedoxin peroxidase in antimony-resistant and –susceptible *Leishmania braziliensis* and *Leishmania infantum* lines

**DOI:** 10.1186/1756-3305-7-406

**Published:** 2014-08-29

**Authors:** Juvana M Andrade, Silvane M F Murta

**Affiliations:** Centro de Pesquisas René Rachou/FIOCRUZ, Avenida Augusto de Lima 1715, Belo Horizonte, 30190-002 MG Brazil

**Keywords:** *L. braziliensis*, *L. infantum*, Drug resistance, Potassium antimony tartrate, Cytosolic tryparedoxin peroxidase, Antioxidant defence

## Abstract

**Background:**

Tryparedoxin peroxidase (TXNPx) participates in defence against oxidative stress as an antioxidant by metabolizing hydrogen peroxide into water molecules. Reports suggest that drug-resistant parasites may increase the levels of TXNPx and other enzymes, thereby protecting them against oxidative stress.

**Methods:**

In this study, the gene encoding cytosolic TXNPx (cTXNPx) was characterized in lines of *Leishmania (Viannia) braziliensis* and *Leishmania (Leishmania) infantum* that are susceptible and resistant to potassium antimony tartrate (Sb(III)). We investigated the levels of mRNA and genomic organization of the *cTXNPx* gene. In addition, we transfected the *Leishmania* lines with the *cTXNPx* gene and analysed the susceptibility of transfected parasites to Sb(III) and to hydrogen peroxide (H_2_O_2_).

**Results:**

Northern blot and real-time reverse transcriptase polymerase chain reaction analyses revealed that the level of *TXNPx* mRNA was approximately 2.5-fold higher in the Sb(III)-resistant *L. braziliensis* line than in the parental line. In contrast, no significant difference in *cTXNPx* mRNA levels between the *L. infantum* lines was observed. Southern blot analyses revealed that the *cTXNPx* gene is not amplified in the genome of the Sb(III)-resistant *Leishmania* lines analysed. Functional analysis of cTXNPx was performed to determine whether overexpression of the enzyme in *L. braziliensis* and *L. infantum* lines would change their susceptibility to Sb(III). Western blotting analysis showed that the level of cTXNPx was 2 to 4-fold higher in transfected clones compared to non-transfected cells. Antimony susceptibility test (EC_50_ assay) revealed that *L. braziliensis* lines overexpressing cTXNPx had a 2-fold increase in resistance to Sb(III) when compared to the untransfected parental line. In addition, these clones are more tolerant to exogenous H_2_O_2_ than the untransfected parental line. In contrast, no difference in Sb(III) susceptibility and a moderate index of resistance to H_2_O_2_ was observed in *L. infantum* clones overexpressing cTXNPx.

**Conclusion:**

Our functional analysis revealed that cTXNPx is involved in the antimony-resistance phenotype in *L. braziliensis*.

**Electronic supplementary material:**

The online version of this article (doi:10.1186/1756-3305-7-406) contains supplementary material, which is available to authorized users.

## Background

Leishmaniasis refers to a spectrum of diseases caused by different species of protozoan parasites belonging to the genus *Leishmania*. An estimated 12 million people are infected with *Leishmania* parasites and an additional 350 million people are at risk worldwide [1]. The clinical manifestation of the disease depends on genetic factors, the host immune system, and mainly on the parasite species involved [2]. In the New World, *L. (Leishmania) infantum* (syn. *L. (L.) chagasi*) [3] is the causative agent of visceral leishmaniasis, whereas *L. (V.) braziliensis* causes cutaneous and mucocutaneous leishmaniasis [4].

The control of leishmaniasis relies primarily on chemotherapy. The pentavalent antimony-containing compounds (sodium stibogluconate- Pentostam® and N-methyl-glucamine- Glucantime®) have been the first-line drugs for treatment of all forms of the disease for more than 70 years [5]. The mechanism of action of antimony has not been fully elucidated. Studies suggest that Sb(V) inhibits macromolecular biosynthesis in amastigotes, possibly altering energy metabolism by inhibiting glycolysis and the oxidative pathway of fatty acids [5, 6]. Sb(III) is purported to generate disturbances in the thiol redox potential of the parasite by inducing the efflux of intracellular thiols and by inhibiting trypanothione reductase, resulting in cell death by oxidative stress [7].

The drugs used against leishmaniasis have several drawbacks, including toxic side effects, high cost, and the occurrence of antimony-resistant *Leishmania* strains [8]. The resistance to pentavalent antimonials has reached epidemic proportions in Bihar (India), where more than 60% of patients with visceral leishmaniasis were unresponsive to Sb(V) treatment [9]. Even though the mechanism of antimony-resistance in *Leishmania* spp. has been widely studied, many questions remain unanswered [8]. It has been described that resistance involves interplay between uptake, efflux, and sequestration of active molecules [8, 10].

Most parasites, including *Leishmania* spp., are more susceptible to reactive oxygen species than their hosts [8, 11]. To prevent cell damage due to reactive oxygen species (ROS), organisms have developed different antioxidant defence systems [12]. In trypanosomatids, peroxidases display a unique feature in using reducing equivalents derived from trypanothione, a dithiol found exclusively in these protozoa, in contrast to other eukaryotes that utilize glutathione and catalase [13, 14]. The function of these antioxidant enzymes include defence against chemical and oxidative stress, by catalyzing the reduction of hydrogen peroxide and small-chain organic hydroperoxides to water and alcohol, respectively. The combined action of trypanothione reductase, tryparedoxin, and tryparedoxin peroxidase is central to the maintenance of a low concentration of hydrogen peroxide (H_2_O_2_) [12].

Tryparedoxin peroxidase (TXNPx) belongs to the 2-cysteine peroxiredoxin family, and can be grouped according to its compartmentalization to the cytosol or mitochondria [14]. These enzymes are highly conserved and they are present in various *Leishmania* species [15–17]. Recently, our proteomic analyses have revealed that cytosolic TXNPx (cTXNPx) is overexpressed in antimony-resistant *L. braziliensis* and *L. infantum* lines [18]. However, its role in the Sb(III)-resistance phenotype in these *Leishmania* species had not been elucidated. Thus, the aim of the present study was to characterize *TXNPx* in these lines by assessing mRNA levels and genomic organization. In addition, functional analysis of cTXNPx was performed to determine whether its overexpression in *Leishmania* lines would change the susceptibility of the parasites to antimony (Sb(III)) and hydrogen peroxide (H_2_O_2_).

## Methods

### *Leishmania*spp. samples

Promastigote forms of *L.* (*Viannia*) *braziliensis* (MHOM/BR/75/M2904) and *L. (Leishmania) infantum* (syn. *L. (L.) chagasi*) (MHOM/BR/74/PP75) were used in this study. Sb(III)-resistant lines were previously obtained from wild-type *L. braziliensis* and *L. infantum* lines by stepwise increasing the drug pressure with Sb(III) [19]. These resistant lines are 20 and 4-fold less sensitive to SbIII than their respective parental counterparts [19]. Promastigote forms of these *Leishmania* lines were grown in M199 medium, harvested in the logarithmic growth phase, washed in PBS and the parasite pellets were used for DNA, RNA and protein preparations.

### RNA and DNA preparations

Total RNA and genomic DNA from *Leishmania* lines were extracted as previously described [20]. Southern and northern blots were carried out using a protocol previously described [21]. Probes for both assays were prepared by amplification of a 592 bp fragment of *cTXNPx* gene from *L. braziliensis* (TritrypDB accession no. Lbr15.1080) using specific primers (forward primer: 5′-CGGTGACGCCAAAATGAAC- 3′; reverse primer: 5′- CTACACCGTGCTGAAGTAGC- 3′). The corresponding fragment has 87.4% nucleotide sequence identify with the *cTXNPx* gene from *L. infantum*. The PCR product was labelled with [α-^32^P] dCTP using Nick Translation Kit (Invitrogen, Carlsbald, CA) following the manufacturer’s instructions. Blots were hybridized with a ^32^P-labelled *cTXNPx*-specific probe, according to Murta *et al*. [21]. Band intensities were analyzed using the software CP ATLAS 2.0 (http://lazarsoftware.com/download.html).

### Quantitative real time RT-PCR

The protocol employed for the preparation of first strand cDNA and the procedure for real time RT-PCR were as previously described [10]. cDNA was used for RT-qPCR amplification on an ABI Prism 7500 - Sequence Detection System (PE Applied Biosystems, Foster City, CA, U.S.A.). The specific primers (forward primer: 5′ CGGTGACGCCAAAATGAAC 3′; reverse primer 5′- GAAGTCAAGCGGGTAGAAGAAGAG- 3′) employed were designed from the complete nucleotide sequence of the *cTXNPx* gene (Lbr15.1080). The 18S small subunit ribosomal RNA (*18S SSU rRNA*) constitutive gene from *Leishmania* was used to normalize the amount of sample analyzed. The primers (forward primer: 5′- TCTAGGCTACCGTTTCGGCTT-3′; reverse primer: 5′-CACACACCGAACCGAAGTTG-3′) were designed from the complete nucleotide sequence of the *18S SSU rRNA* gene (*LmjF.27.rRNA.01*). Both pair of primers amplified fragments of 136 bp and 97 bp respectively, in all *Leishmania* lines (data not shown). Standard curves were prepared for each run using known quantities of pCR 2.1-TOPO plasmids (Invitrogen) containing the *cTXNPx* and *18S SSU rRNA* genes. Estimates of transcript levels were obtained using the Sequence Detection System data analysis software. Values were normalized to those obtained for *18S SSU rRNA* for each sample.

### Generation of cTXNPX overexpressing lines

A 600 bp fragment corresponding to *L. braziliensis* cTXNPx ORF (TritrypDB accession number LbrM15.1080) was amplified with *Pfx* DNA polymerase (Invitrogen) from *L. braziliensis* genomic DNA using the forward primer: 5′-t*AGATCT*ccaccATGTCCTGCGGTGACGCCAA-3′ and the reverse primer: 5′-tt*AGATCT*CTACACCGTGCTGAAGTAGC-3′ in which the italicized sequences correspond to *BgI*II restriction site. The obtained PCR product was cloned into the pGEMT-easy vector (Invitrogen) and subsequently submitted on an ABI 3130 (Applied Biosystems, Foster City, CA, USA) for confirmation of correct sequence. The pGEM-LbcTXNPX construct was restricted with *Bgl*II and the released fragment was subcloned into the dephosphorylated pIR1-BSD expression vector, generously provided by Dr. Stephen Beverley (Washington University in St. Louis – USA). To confirm correct direction of cloning, the construct was then digested with *Bam*HI releasing fragments that confirmed the sense direction of gene. Thereafter, the constructs pIR1-BSD (empty vector) and pIR1-BSD-Lb cTXNPx were linearized by *Swa*I digestion and electroporated into *L. braziliensis* and *L. infantum* wild type lines using a GenePulser XCell (BioRad, Hercules, CA, USA). This allowed integration of the vector into the ribosomal small subunit locus [22]. Colonies were obtained following plating on semisolid M199 medium containing Blasticidin (BSD) (10 μg/ml), after 1–2 weeks. Clonal lines were generated and the presence of construct was confirmed by PCR tests using genomic DNA with specific primers for the BSD marker.

### Western blotting analysis

In order to investigate the cTXNPx levels of transfected lines, Western blot assays were carried out. Total protein from the different *Leishmania* clonal lines were extracted according to the protocol described by Gamarro *et al.* [23]. Proteins extracts (20 μg) were separated by electrophoresis on a 12% SDS polyacrylamide gel and electrotransferred onto nitrocellulose membrane (Bio-Rad). The membrane was blocked by incubation with 5% instant non-fat dry milk in PBS supplemented with 0.05% Tween 20 (PBS-T) for 1 h. The membrane was then washed twice in PBS-T for 5 min and incubated for 16 h at 4°C in the blocking solution with a polyclonal rabbit anti-*T. cruzi* TXNPx antibody (1:500) [24] (kindly provided by Dr. Fernanda Nogueira, CPqRR, Belo Horizonte, Brazil). The blots were washed three times in PBS-T and then incubated for 1 h with alkaline phosphatase-conjugated anti-rabbit IgG (Invitrogen) diluted 1:6.000 in blocking solution. Subsequently, the blots were developed using a colorimetric method (Bio-Rad) following the manufacturer’s instructions. The blots were normalized using a monoclonal anti-α-tubulin antibody (1:10.000) (Sigma, St. Louis, USA). The intensity of the bands was analyzed using the software CP ATLAS 2.0.

### Susceptibility of *Leishmania*spp. clonal lines to Sb(III) and hydrogen peroxide

Promastigotes of wild-type *L. braziliensis* and *L. infantum* transfected or non-transfected with the constructs pIR1-BSD (empty vector) or pIR1-BSD-LbcTXNPx were submitted to Sb(III) and hydrogen peroxide (H_2_O_2_) susceptibility tests. Parasites were incubated in M199 medium at 2 × 10^6^ cells ml^−1^ in 24-well plates in the absence or presence of various concentrations of SbIII (0.0125 to 1 mg/ml) or H_2_O_2_ (200 to 600 μM) for 48 hours. The concentration of Sb(III) or H_2_O_2_ required to inhibit the growth by 50% (EC_50_) was determined using a Z1 Coulter Counter (Beckman Coulter, Fullerton, CA, USA). EC_50_ values were determined from three independent measurements, each performed in triplicate, using the linear interpolation method [25].

### Statistic analysis

All experiments were performed at least three times and data have been represented as mean ± standard deviation. Data were analyzed by Student’s *t* test performed using the software GraphPad Prism 5.0. A *p* value of less than 0.05 was considered statistically significant.

## Results

### Genomic organization of the *cTXNPX*gene

Genomic organization of the *cTXNPx* gene in Sb(III)-resistant and -susceptible lines of *L. braziliensis* and *L. infantum* was determined by Southern blot analysis of parasite DNA digested with an endonuclease (*Eco*RI or *Bam*HI). Hybridization of the blots with an LbcTXNPx gene specific probe revealed that *Eco*R*I*-digested DNA gave a major band of 14.0 kb for both *L. braziliensis* lines, and bands of 0.6, 0.8, 4.0, and 14.0 kb for *L. infantum* lines (Additional file 1: Figure S1). Upon hybridization, *Bam*HI-digested DNA from *L. braziliensis* lines identified a single 12 kb band, while lines from *L. infantum* contained bands of 0.5, 0.6, and 12 kb (Additional file 1: Figure S1). Others bands having a low intensity were also observed. We observed a polymorphism in the *cTXNPx* sequence between both *Leishmania* species analysed. Comparative densitometry of the bands showed no amplification of *cTXNPx* gene in both Sb(III)-resistant *Leishmania* lines.

### Determination of *cTXNPx*mRNA levels

**Figure 1:**
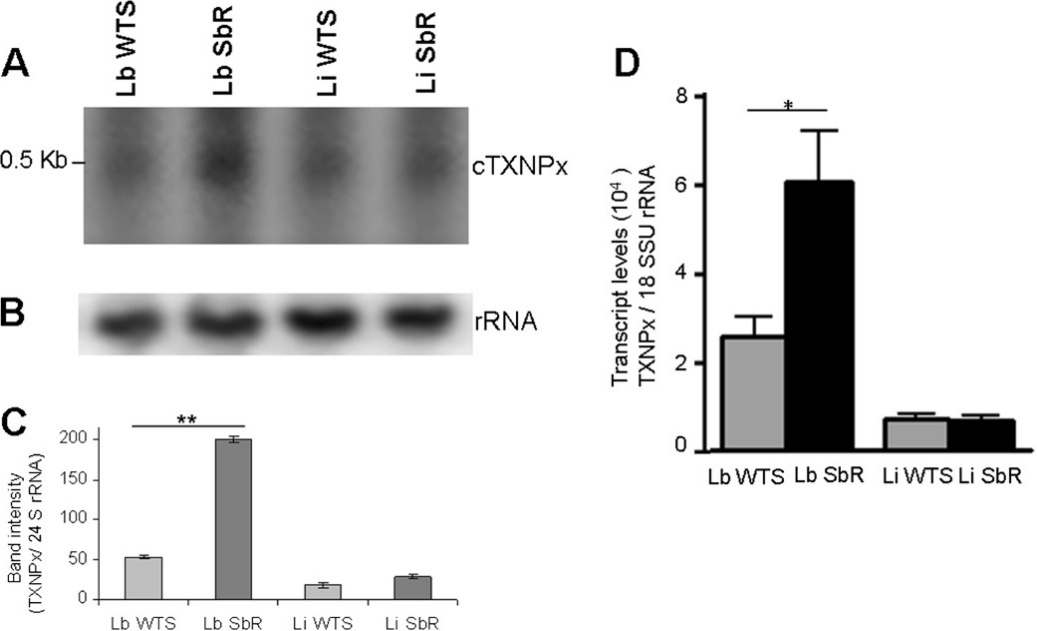
**Levels of** ***cTXNPx*** **gene transcript in Sb(III)-susceptible and Sb(III)-resistant** ***L. braziliensis*** **and** ***L. infantum*** **lines. (A)** Northern blot analysis of total RNA (20 μg) from *Leishmania* spp. lines separated on a 1.2% agarose gel and transferred to nylon membranes. Blots were hybridized with a ^32^P-labelled *cTXNPx*-specific probe. **(B)** As a control, the same nylon membrane was hybridized with a ^32^P-labelled *24S rRNA*-specific probe. **(C)** Quantification of bands was done by densitometric analysis using the software CP ATLAS 2.0. **(D)** Levels of *cTXNPx* mRNA of *L. braziliensis* and *L. infantum* lines determined quantitatively (relative to the 18S small subunit ribosomal RNA -*18S SSU rRNA*) by real-time PCR. Mean values of the transcript levels of *cTXNPx/SSU* ± standard deviations as determined from three independent experiments are shown. Statistically different values are indicated as follows: *p < 0.04, **p < 0.0002.

The levels of *cTXNPx* mRNA across different parasite lines were evaluated by Northern blot. A transcript of 0.5 kb was detected in Northern blots from Sb(III)-susceptible and -resistant *L. braziliensis* and *L. infantum* lines following hybridization with a ^32^P-labelled cytosolic tryparedoxin peroxidase from *L. braziliensis* (*LbcTXNPx*) gene specific probe (Figure 1A). Loading controls using a ribosomal RNA probe are shown in Figure 1B. Densitometry of the transcript profiles revealed that the *cTXNPx* mRNA level was 2.5-fold higher in the Sb(III)-resistant *L. braziliensis* line when compared to the parental line (Figure 1C). No difference in the levels of *cTXNPx* mRNA was detected in both Sb(III)-resistant and -susceptible *L. infantum* lines.

*cTXNPx* mRNA levels were determined with greater precision by RT-qPCR. The amount of *cTXNPx* cDNA and *18S SSU rRNA* in different *Leishmania* lines was determined by linear regression analysis using the PCR threshold cycle (C_T_) values obtained from the standard curve generated with known amounts of the plasmids containing these genes. The amount of *cTXNPx* cDNA in each line was normalized to the reference housekeeping gene, *18S SSU rRNA* (LmjF.27.rRNA.01). The results confirmed the northern blot data, demonstrating that the level of *cTXNPx* gene transcripts was 2.5-fold higher in the Sb(III)-resistant *L. braziliensis* line when compared to the parental line. In addition, no difference was detected between the lines of *L. infantum* analysed (Figure 1D).

### Overexpression of *cTXNPx*gene in *L. braziliensis*and *L. infantum*lines

**Figure 2:**
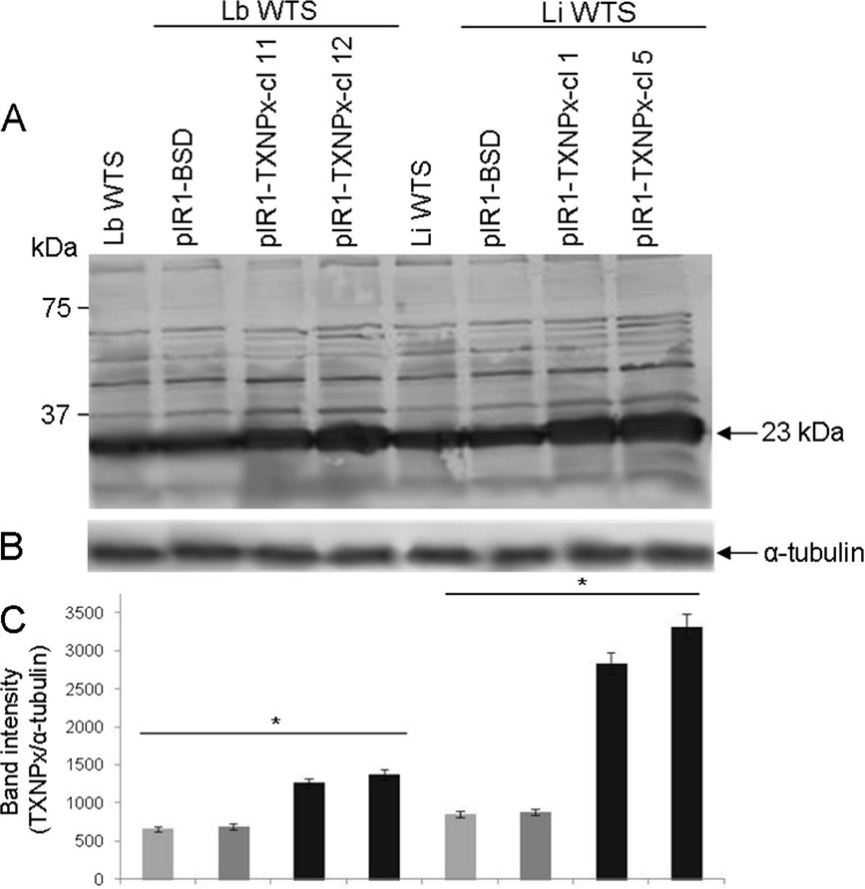
**cTXNPx expression levels in clonal lines from** ***L. braziliensis*** **and** ***L. infantum*** **untransfected or transfected with constructs pIR1-BSD/pIR1-BSD-LbcTXNPx.** Total protein (20 μg) was separated on a 12% SDS polyacrylamide gel and blotted onto nitrocellulose membranes. The blots were probed with a polyclonal rabbit anti-*T. cruzi* cTXNPx antibody (1:500) **(A)** and with a monoclonal anti-α-tubulin antibody (1:10.000) **(B)** and developed with NBT/BCIP. **(C)** Quantification of bands was done by densitometric analysis using the software CP ATLAS 2.0. Statistically different values are indicated as follows: *p < 0.003.

We transfected wild-type *L. braziliensis* and *L. infantum* lines with the construct pIR1-BSD-LbcTXNPx to generate transfectants overexpressing cTXNPx. Linearization of the vector allowed integration of the construct into the ribosomal small subunit locus, by homologous recombination [26]. The successful integration of constructs was confirmed by PCR, using genomic DNA as template and with specific primers for the Blasticidin (BSD) marker. About 24 clones each for pIR1-BSD (empty vector) and pIR1-BSD-LbcTXNPX from *L. braziliensis* and *L. infantum* lines were analysed by PCR. It was observed that all blasticidin-resistant clones produced a fragment of 399 bp, indicative for the BSD marker (data not shown). These clones were subjected to Western blotting analysis to evaluate the level of cTXNPX. The anti-*T. cruzi* TXNPX antibody [24] recognized a 23 kDa band in all *Leishmania* clones (Figure 2A). Densitometry of the cTXNPx band using an anti-α tubulin antibody as reference (Figure 2B) showed that the level of cTXNPx was 2 to 4-fold higher in the transfected clones from both *L. braziliensis* and *L. infantum* lines when compared to the untransfected controls (Figure 2C).

### Susceptibility of cTXNPX overexpressing lines to Sb(III)

**Figure 3:**
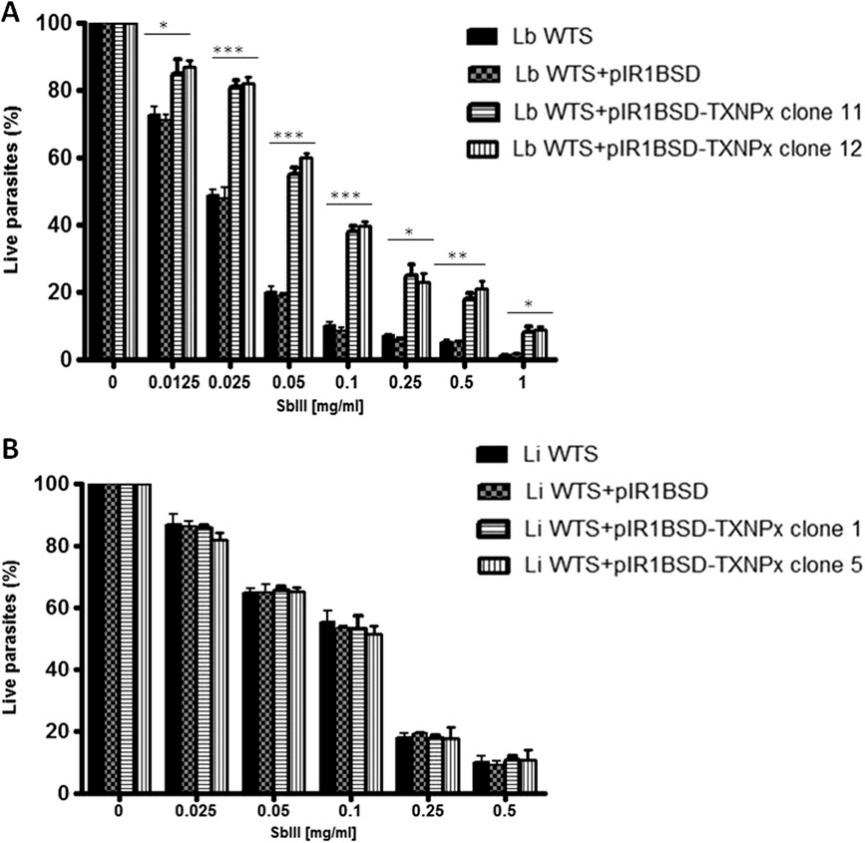
**Sb(III) susceptibility of clonal lines from** ***L. braziliensis*** **(A) and** ***L. infantum*** **(B) untransfected or transfected with constructs pIR1-BSD or pIR1-BSD-LbcTXNPx.** Parasites were incubated in M199 medium in the absence or presence of various concentrations of Sb(III) (0.0125 to 1 mg/ml) for 48 hours and the percentages of live parasites were determined using a Z1 Coulter Counter. Mean values ± standard deviations from three independent experiments in triplicate are shown. Statistically different values are indicated as follows: *p < 0.04, **p < 0.007 and ***p < 0.002.

In order to investigate whether overexpression of cTXNPx gene favours an antimony-resistance phenotype, clonal lines from *L. braziliensis* and *L. infantum* transfected with the constructs pIR1-BSD (empty vector) or pIR1-BSD-LbcTXNPx and untransfected parasites were subject to a Sb(III) susceptibility test. As shown in Figure 3A, with increasing concentrations of Sb(III) there was a rapid decline in the percentage of live parasites in both untransfected and empty vector transfected *L. braziliensis* lines when compared to cTXNPx overexpressing lines. The concentration of Sb(III) required to inhibit the growth of the parasites by 50% (effective concentration- EC_50_) was 0.03 mg/ml for both controls. In contrast, cTXNPx overexpressing lines were 2.3-fold more resistant to Sb(III), with an EC_50_ of 0.07 mg/ml. Interestingly, *L. infantum* cTXNPx overexpressing lines did not show an increase in resistance towards Sb(III). The cTXNPx overexpressing lines had an EC50 value (0.11 mg/ml) similar to that of the controls (0.12 mg/ml) (Figure 3B).

### Tolerance of cTXNPx overexpressing lines to hydrogen peroxide

**Figure 4:**
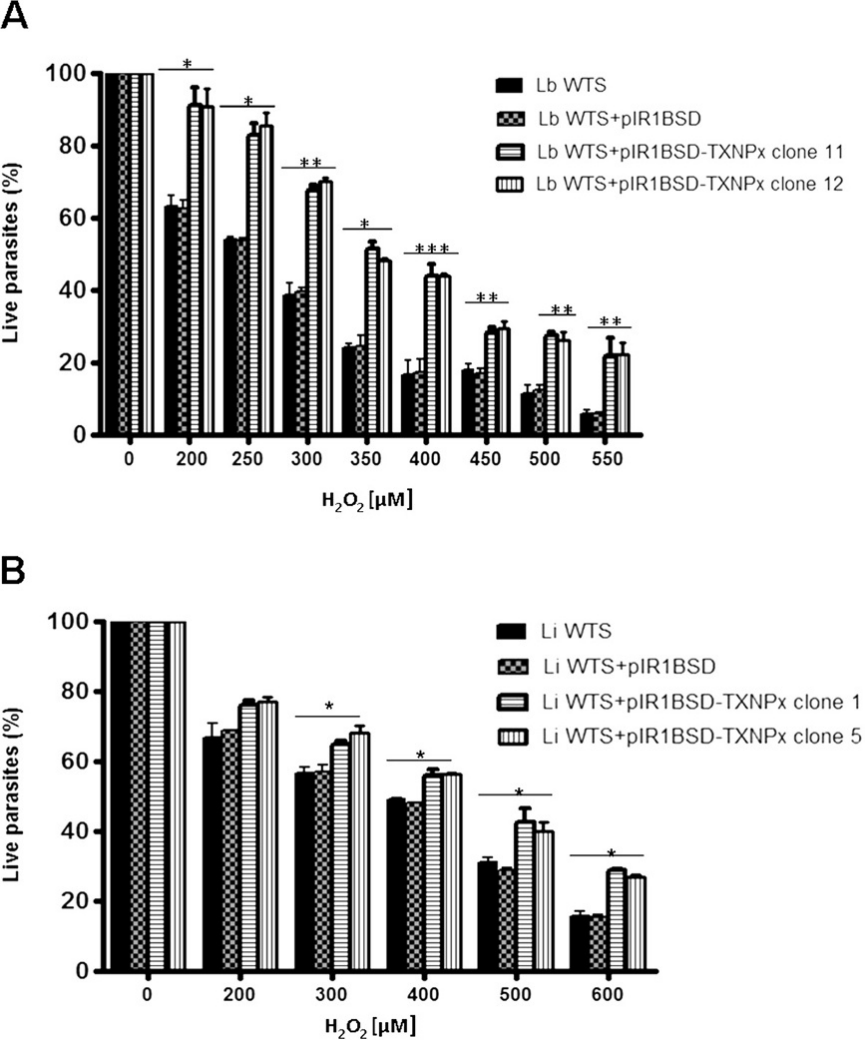
***In vitro*** **tolerance of** ***L. braziliensis*** **(A) and** ***L. infantum*** **(B) lines untransfected or transfected with constructs pIR1-BSD/pIR1BSD-cTXNPx, to exogenous hydrogen peroxide.** Parasites were cultured for 48 h in the presence of different concentrations of hydrogen peroxide and the percentages of live parasites were determined using a Z1 Coulter Counter. Mean values ± standard deviations from three independent experiments in triplicate are shown. Statistically different values are indicated as follows: *p < 0.04, **p < 0.005 and ***p < 0.0001.

The tolerance to oxidative stress generated by increased concentrations of hydrogen peroxide was evaluated in the cTXNPx overexpressing lines of *L. braziliensis* and *L. infantum* (Figure 4)*. In vitro* assays revealed that the cTXNPx overexpressing LbWTS clones 11 and 12 displayed an EC_50_ value towards H_2_O_2_ of 408 and 400 μM, respectively (Figure 4A). In contrast, untransfected and empty vector transfected lines exhibited lower EC_50_ values for H_2_O_2_ (260 and 256 μM, respectively). Thus, cTXNPx overexpressing lines were 1.56-fold (*p* < 0.001) more tolerant to exogenous hydrogen peroxide than controls in *L. braziliensis.* A moderate index of resistance to H_2_O_2_ was observed for the cTXNPx overexpressing lines of *L. infantum* compared to that of *L. braziliensis.* LiWTS clones 1 and 5 displayed EC_50_ values of 456 and 450 μM, respectively towards H_2_O_2_ (Figure 4B). In contrast, untransfected and empty vector transfected lines exhibited lower EC_50_ values for H_2_O_2_ (373 and 368 μM, respectively). Thus, cTXNPx overexpressing lines were 1.22-fold (*p* < 0.001) more tolerant to exogenous hydrogen peroxide than controls in *L. infantum.*

## Discussion

Antioxidant defence is a promising target for chemotherapy against trypanosomatids, since these organisms present a unique mechanism for detoxification of peroxides that is dependent on trypanothione, which differs from the glutathione-based system found in vertebrates. In trypanosomatids, TXNPx participates in defence against oxidative stress by metabolizing hydrogen peroxide into water molecules [12]. This enzyme is critical to the survival of *Leishmania* during oxidative stress generated by macrophages and by drugs [27]. In previous studies carried out by our group, using proteomic analysis it was indicated that seven protein spots corresponding to TXNPx were 2 to 5-fold more abundant in the Sb(III)-resistant lines of both *L. braziliensis* and *L. infantum* species than in their Sb(III)-susceptible parental lines [18]. In the present work, we have extended these results by characterizing cTXNPx in Sb(III)-susceptible and -resistant *L. braziliensis* and *L. infantum* lines and by performing functional analysis of this enzyme.

Our results demonstrated that in the Sb(III)-resistant *L. braziliensis* line the increase in *cTXNPx* mRNA levels (2.5-fold) is correlated with high cTXNPx protein levels (3-fold; Matrangolo *et al*. [18]). In contrast, no difference in the transcription level of this gene was found for Sb(III)-resistant and -susceptible *L. infantum* lines. However, Matrangolo *et al*. [18] showed that the cTXNPx protein level was 1.6-fold higher in the Sb(III)-resistant *L. infantum* lines when compared with Sb(III)-susceptible parental lines. Since gene expression in trypanosomatids is regulated mainly at the post-transcription level [28], our results suggest that the higher levels of cTXNPx protein detected in the Sb(III)-resistant *L. infantum* (LiSbR) line maybe related to an increased stability of mRNA or more efficient protein translation when compared with the wild-type *L. infantum* (LiWTS) pair.

Altogether, these findings are in agreement with existing data reporting an increase in *TXNPx* mRNA and TXNPx protein levels in drug-resistant parasites. Nogueira *et al*. [24] demonstrated an increased expression of *TXNPx* transcript and TXNPx protein in *T. cruzi* resistant to benznidazole. Metronidazole-resistant *Entamoeba histolytica* showed a 3-fold increase in its *TXNPx* mRNA levels [29]. In an Sb(III)-resistant *L. tarentolae* line, an increase of cytosolic (6.5-fold) and mitochondrial (1.8-fold) TXNPx enzymes has been reported [30]. Protein analyses indicated high levels of TXNPx in antimony-resistant *L. donovani* lines [31] and gentamicin-resistant *L. infantum* clones [32].

In order to investigate the role of cTXNPx in protecting the parasite against oxidative stress and its involvement in Sb(III)-resistance, this enzyme was overexpressed in *L. braziliensis* and *L. infantum* promastigotes. Interestingly, clones from *L. braziliensis* that overexpress the cTXNPx were 2-fold more resistant to Sb(III). This result indicates that the enzyme is involved in the Sb(III)-resistance phenotype probably along with other enzymes, since the drug resistance phenotype is known to be multifactorial and multigenic. TXNPx is a key antioxidant enzyme important for parasite resistance to oxidative stress. Previous studies have demonstrated that Sb(III) perturbs the thiol redox potential of the parasite, leading to accumulation of reactive oxygen species (ROS) [7, 33]. Sb(III) forms a complex with either trypanothione or glutathione that can be sequestrated in an intracellular compartment or directly excreted from parasites in response to SbIII treatment [7, 8]. Thus, Sb(III) decreases intracellular thiol buffer capacity, and it also increases the intracellular concentration of the disulfide forms of these thiols through inhibition of trypanothione reductase [7]. These effects of Sb(III) favour increased levels of ROS. Overexpression of TXPNx confers resistance to Sb(III) by an increased enzyme activity that acts to reduce levels of ROS induced by exposure to Sb(III). Data from literature reinforce our results in *L. braziliensis,* since overexpression of TXNPx in *L. tarentolae* caused a significant increase in resistance to Sb(III) [30]. In contrast, overexpression of an enzymatically inactive TXNPx failed to result in resistance to Sb(III) [30]. These data suggest that the mechanism of TXNPx-dependent resistance is likely due to enhanced antioxidant activity.

Reports have shown that parasites overexpressing cTXNPx exhibit a high level of resistance to reactive oxygen radicals. In *T. cruzi,* it has been reported that overexpression of TXNPx protects the parasite from H_2_O_2_ and organic peroxide *t*-butyl hydroperoxide damage [34, 35]. Lyer *et al*. [36] observed an increase in the cTXNPx levels in *L. donovani* after exposure to H_2_O_2_. Additionally, the authors also demonstrate that *L. donovani* parasites transfected with cTXNPx are more resistant to antimony and exhibit an increase in virulence when compared to parental parasites. All these data are in agreement with our results showing that cTXNPX overerexpressing *L. braziliensis* and *L. infantum* clones are more tolerant to exogenous H_2_O_2_ than an untransfected parental line. However, the *L. infantum* clones present a moderate index of tolerance to H_2_O_2_ when compared to *L. braziliensis*. This difference could explain, at least in part, the absence of resistance to Sb(III) in these clones, since our results show that overexpression of this enzyme has no direct involvement in the Sb(III)-resistance in *L. infantum*. In addition, the absence of Sb(III) resistance in cTXNPx overerexpressing *L. infantum* could also be due to differences in antimony-resistance mechanisms between these two *Leishmania* species. Moreira *et al.* [10] demonstrated that an Sb(III)-resistant *L. braziliensis* line presented an increased expression of the MRPA gene product and a reduction in the accumulation of antimony. However, no difference was detected between the Sb(III)-resistant and susceptible *L. infantum* lines.

## Conclusion

The results of the functional analysis revealed that cTXNPx is involved in the antimony-resistance phenotype in *L. braziliensis*. However, in *L. infantum,* this enzyme does not seem to be directly associated with resistance to Sb(III). Interestingly, Wyllie *et al.* [31] have reported elevated levels of TXNPx in antimony-unresponsive *L. donovani* field isolates. These data suggest that increased expression of this enzyme may play an important role in clinical resistance to antimony.

## Electronic supplementary material

Additional file 1: Figure S1: Southern blot analysis of the *cTXNPx* gene from wild-type and SbIII-resistant *L. braziliensis* and *L. infantum* lines. Genomic DNA (10 μg) was digested with *Eco*RI (a) and *Bam*HI (b) endonucleases, subject to electrophoresis on a 1% agarose gel and transferred to nylon membranes. Blots were hybridized with a ^32^P-labeled *cTXNPx*-specific probe. As control, the same nylon membranes were hybridized with a ^32^P-labeled rRNA-specific probe (c and d). The molecular weight markers used were the 1 Kb Plus DNA ladder. (DOC 292 KB)

## Abbreviations

SbIII: Potassium antimonyl tartrate
WTS: Wild-type susceptible
SbR: SbIII-resistant
Lb: *L. (V.) braziliensis*
Li: *L. (L.) infantum*
cTXNPx: Tryparedoxin peroxidase cytosolic
qRT-PCR: Real time PCR
ROS: Reactive oxygen species
H2O2: Hydrogen peroxide
EC: Effective concentration.
