# Supplementary material for: Functional analysis of cytosolic tryparedoxin peroxidase in antimony-resistant and –susceptible Leishmania braziliensis and Leishmania infantum lines
Source: Parasit Vectors. 2014 Aug 29;7:406. doi: 10.1186/1756-3305-7-406 (PMC4261743; doi:10.1186/1756-3305-7-406)
Supplement: Supplementary file 1 — Additional file 1: Figure S1: Southern blot analysis of the cTXNPx gene from wild-type and SbIII-resistant L. braziliensis and L. infantum lines. Genomic DNA (10 μg) was digested with EcoRI (a) and BamHI (b) endonucleases, subject to electrophoresis on a 1% agarose gel and transferred to nylon membranes. Blots were hybridized with a 32P-labeled cTXNPx-specific probe. As control, the same nylon membranes were hybridized with a 32P-labeled rRNA-specific probe (c and d). The molecular weight markers used were the 1 Kb Plus DNA ladder. (DOC 292 KB) [file 13071_2014_1594_MOESM1_ESM.doc]

**ADDITIONAL FILE 1**

**Functional analysis of cytosolic tryparedoxin peroxidase in antimony-resistant and –susceptible *Leishmania braziliensis* and *Leishmania infantum* lines**

Juvana M. Andrade, Silvane M. F. Murta

**
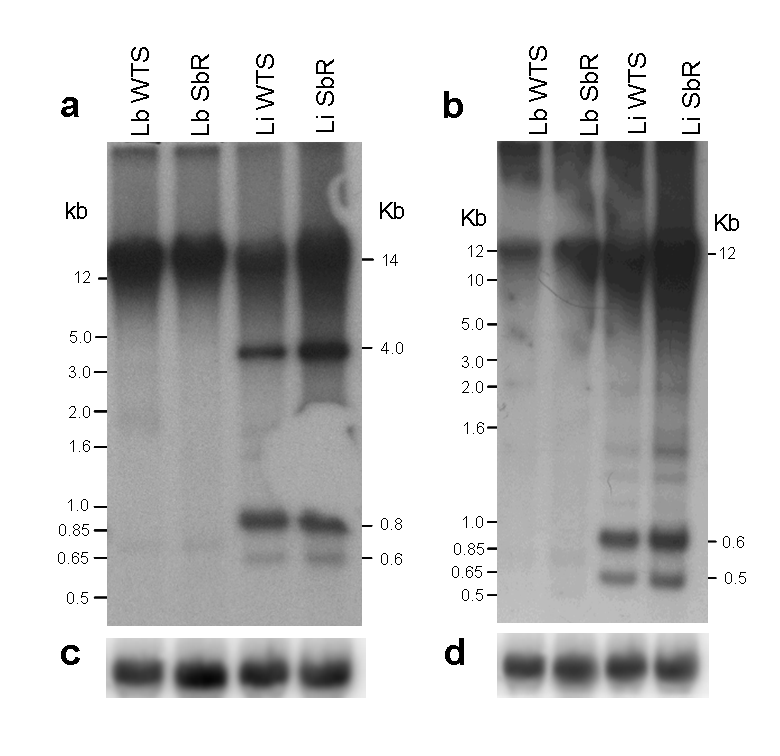
**

**Figure S1: Southern blot analysis of the *cTXNPx* gene from wild-type and SbIII-resistant *L. braziliensis* and *L. infantum* lines.** Genomic DNA (10 µg) was digested with *Eco*RI (**a**) and *Bam*HI (**b**) endonucleases, subject to electrophoresis on a 1% agarose gel and transferred to nylon membranes. Blots were hybridized with a ^32^P-labeled *cTXNPx*-specific probe. As control, the same nylon membranes were hybridized with a ^32^P-labeled *rRNA*-specific probe (**c** and **d**). The molecular weight markers used were the 1 Kb Plus DNA ladder.
